# Simplified, High Yielding Extraction of Xylan/Xylo-Oligosaccharides from *Palmaria palmata*: The Importance of the Algae Preservation Treatment

**DOI:** 10.3390/md23080302

**Published:** 2025-07-29

**Authors:** Diogo Coelho, Diogo Félix Costa, Mário Barroca, Sara Alexandra Cunha, Maria Manuela Pintado, Helena Abreu, Margarida Martins, Tony Collins

**Affiliations:** 1 Centre of Molecular and Environmental Biology (CBMA)/Aquatic Research Network (ARNET), Department of Biology, University of Minho, 4710-057 Braga, Portugal; diogo@bio.uminho.pt (D.C.); pg40135@alunos.uminho.pt (D.F.C.); mariobarroc@gmail.com (M.B.); 2 Institute of Science and Innovation for Bio-Sustainability (IB-S), University of Minho, 4710-057 Braga, Portugal; 3 Universidade Católica Portuguesa, CBQF—Centro de Biotecnologia e Química Fina—Laboratório Associado, Escola Superior de Biotecnologia, Rua Diogo Botelho 1327, 4169-005 Porto, Portugal; scunha@ucp.pt (S.A.C.); mpintado@ucp.pt (M.M.P.); 4 Independent Seaweed Consultant, 3870-322 Murtosa, Portugal; helena@algalink.eu; 5 ALGAplus, 3830-352 Ílhavo, Portugal; margarida.martins@algaplus.pt

**Keywords:** xylan, *Palmaria palmata*, algae pretreatment, process temperature, extraction time

## Abstract

The complex plant cell wall heteropolysaccharide xylan, and its breakdown products xylo-oligosaccharides and xylose, are value-added compounds with a plethora of potential applications in diverse areas. They are nonetheless currently poorly exploited, with a major bottleneck being the unavailability of efficient, low-cost, high-yield production processes. The major objective of the present study is to identify and characterise a high-yield process for the preparation of highly pure xylan/XOS products from the macroalga *Palmaria palmata*. Currently, most xylan is extracted from land-sourced lignocellulosic feedstocks, but we take advantage of the high xylan content, xylan aqueous solubility, lignin-free nature, weakly linked cell wall matrix, and sustainability of the macroalga to identify a simple, sustainable, high-yield, novel-xylan-structure extraction process. This is composed of five steps: alga oven drying, milling, aqueous extraction, centrifugation, and dialysis, and we show that the alga preservation step plays a critical role in component extractability, with oven drying at high temperatures, ~100 °C, enhancing the subsequent aqueous extraction process, and providing for xylan yields as high as 80% of a highly pure (~90%) xylan product. The process developed herein and the insights gained will promote a greater availability of these bioactive compounds and open up their application potential.

## 1. Introduction

Xylan is a complex plant cell wall polysaccharide that is important for plant structure, cohesion, strength, and defence [1,2]. It is found in a wide variety of plants, including hardwoods, softwoods, grasses (e.g., cereals, sugar cane) [1,3] and some macroalgae [4,5]. Its structure varies considerably, being characterised by a backbone chain of β-D-xylopyranosyl units which, depending on the plant species, growth stage, and tissue, may have various degrees of polymerisation, be linked by either β-1,4 or/and β-1,3-bonds, and be non-substituted or substituted to varying degrees by various sidechain groups [3,6,7]. Land plant xylans are typically composed of a β-1,4-linked backbone, which is variably substituted with, among others, various glucuronyl, methyl-D-glucuronyl, α-L-arabinosyl, acetyl, feruloyl, and/or p-coumaroyl groups. In contrast, macroalgae contain a β-1,3-linked or mixed β-1,3/β-1,4-linked, linear, non-substituted xylan [3,4,5,7].

Xylan, and in particular its hydrolysis products, xylo-oligosaccharides (XOS) and xylose, have numerous potential applications in various areas [8,9]. Xylan has been shown to have potential for example in food and feed (e.g., dietary fibre) [10,11,12], therapeutic (e.g., drug/gene delivery, tissue engineering, and gut health) [13,14,15,16], materials (e.g., packaging, bioadsorbents, biomaterials, and biocomposites) [8,17,18,19,20], chemicals (e.g., solvents and dispersants) [21,22], and textiles (e.g., fibre modification, functional coating, and reinforcement) applications [23], as well as in research and development (as a substrate in xylanolytic enzymes studies) [24,25]. XOS are soluble, low molecular weight xylose-based oligomers derived from xylan; they are most commonly defined as being composed of a backbone of two to ~ten β-D-xylopyranosyl units, but higher degrees of polymerisation (up to sixty) have also been reported [26]. These have already been shown to be safe, effective, and stable prebiotics, being beneficial for gastrointestinal health [27,28]. Importantly, they have also been shown to display anti-cancer [29], anti-oxidant [30], anti-inflammatory [31], immunostimulatory [31], and/or anti-microbial activities [32], while applications in diabetes treatment/prevention [33,34], and as low-calorie sweeteners [35] have also been reported. Finally, xylose, the monomer of xylan and XOS, has enormous potential as a platform intermediate for the manufacture of a plethora of industrial, food, and pharmaceutical products [36,37,38]. It can be converted to products and precursors such as furfural, xylonic acid (recognised as a top 30 building block chemical), isopropanol, ethanol, and xylitol, with these being employed in the production of various products including fuels, antifreeze products, heat-transfer agents, resins, plastics, copolyamides, preserving agents, emulsifiers, pesticides, cement retardants, and even sweeteners and acidulants [36,37,38].

Even though the value of xylan, XOS and xylose is clearly understood, these compounds remain underexploited. Presently, they are almost exclusively isolated from land-sourced lignocellulosic feedstocks via multi-step processes employing various hazardous chemicals, harsh physical treatments, and/or enzyme(s) hydrolysis [39,40,41,42,43]. These processes are often characterised by environmental and/or safety issues, the production of toxic side products and/or inhibitors that can interfere with downstream processes, and/or the need for additional purification steps and/or specialised equipment, and high costs, which, ultimately, lead to limited exploitation [39,40,41,42,43,44,45]. Interestingly, macroalgae have the potential to address, at least partially, these limitations, as a number of red and green macroalgae are rich in xylan and absent of lignin, a complex organic polymer that interferes with extraction procedures [4,46]. Additionally, macroalgae are a fast-growing, abundant, and carbon-neutral renewable marine resource that do not compete with traditional food crops for space or resources (arable land and freshwater) and, in fact, have several benefits over traditional land-based crops in terms of productivity, nutritional value, and environmental impact.

*Palmaria palmata* is a Rhodophyta commonly found on North Atlantic shores (latitude of ~40–80° N) that can be sustainably produced by aquaculture approaches [47], including integrated multi-trophic aquaculture (IMTA) [48,49]. It is composed of up to 35% xylan (by dry weight), with the structure of this xylan (i.e., non-substituted, β-1,3/β-1,4-linked) being distinct from currently used land plant xylans, and hence pointing to novel characteristics, functions, and potentially also, novel applications for this xylan source. Protein (up to 35%) and ash (up to 40%) are the other major components of this alga, with only reduced amounts of other components (galactose, glucose, phenolics, and lipids) having been reported [49,50]. Furthermore, the xylan present has been shown to be water soluble and to be weakly held in the cell wall matrix [46], and this, in combination with the absence of lignin and low levels of other constituents, allowed us to develop a simple, chemical-free method for the high-yield extraction of xylo-oligosaccharides from this red macroalga. In particular, we show that the drying temperature used in the first step of alga treatment following cultivation, i.e., the biomass preservation step, critically affects the downstream extraction and fractionation of various components. Herein, the fine-tuning of this preservation step facilitated simplified aqueous extraction of high yields of highly pure xylan. This work will unlock the exploitation potential of xylan and its hydrolysates, promoting greater availability, more widespread use, and development into new application areas.

## 2. Results and Discussion

A current major bottleneck to the more widespread employment of xylan and its hydrolysates (XOS and xylose) in industry is the lack of large quantities of these compounds at high purity. In this study, we attempted to address this limitation by taking advantage of the high xylan content and solubility of this xylan in water, the lignin-free nature, weakly linked cell wall matrix, and sustainability of the macroalga *P. palmata*, to develop a simplified, more environmentally friendly approach for high yields of highly pure xylan/XOS.

### 2.1. Compositional Analysis of P. palmata

The composition of *P. palmata* is known to vary with the strain used, the production and harvesting conditions, and geographical and seasonal variations [51]. Therefore, as a first step in our study, we determined the composition of the *P. palmata* batch used. This was found to be principally composed of ash (28.3 ± 0.13%), xylan (25.8 ± 0.12%), and protein (25.6 ± 0.67%), with small amounts of galactose (3.74 ± 0.2%), lipids (1.6 ± 0.1%), phenolic compounds (0.9 ± 0.05%), and glucose (0.23 ± 0.02%) being also detected. No insoluble glucans were detected with the method employed. This composition is in agreement with previous studies where values in the ranges of 10–40% (ash), 20–35% (xylan), 10–35% (protein), 3–16% galactose, 0.4–4% (lipids), 0.5–1% (phenolic compounds), and ~2% glucose, have been reported [48,51,52,53,54,55,56,57,58].

### 2.2. Algae Preservation: Drying

In common with other macroalgae, the fresh *P. palmata* used in our study was also found to have a high water content, at ~90%. Such a high water content increases the susceptibility of the macroalga to microbial decomposition once harvested, while also increasing weight and volume, and thereby provoking technical difficulties and increased costs for packaging, storage, and transportation. A number of long-term preservation methods and storage conditions have been developed for macroalgae, including drying, freezing, lyophilisation, wilting, and ensilaging approaches, as well as milling and pressing [59,60,61,62]. Of these, drying to reduce water activity, volume, and weight, in combination with milling to further reduce volume and potentially also enhance component extraction, are commonly used. In particular, low-temperature drying, to conserve heat-sensitive algal components, is commonly used. Interestingly, on the other hand, studies investigating the effects of drying temperature on the functional, structural, and visual characteristics of macroalgae noted an increased polysaccharide extractability for some algae when higher drying temperatures were employed. Silva and colleagues [61] observed a positive correlation between drying temperature and the extractability of ulvans from *Ulva rigida*, and fucoidans from *Fucus vesiculosus*, over the 25–60 °C temperature range examined. Similarly, Tello-Ireland et al. [60] observed increasing agar aqueous extraction from *Gracilaria chilensis* with higher drying temperatures over the 40 to 70 °C range examined. Furthermore, decreased water-holding capacity, rehydration ratio, and firmness with increasing drying temperature were also reported in these studies, probably resulting from algae structure alterations and deformation, matrix degradation, and reduced cell wall integrity. We therefore investigated this in *P. palmata*. In contrast to plant sourced xylans which are generally insoluble or poorly soluble in water, *P. palmata* xylan is water soluble [46,49], and this, in conjunction with the absence of lignin, and weak structural makeup of the macroalga, along with the potential for a decreased structural integrity of the alga following high-temperature drying, prompted us to investigate a combination of alga drying, milling, and aqueous extraction for a simplified, sustainable xylan/XOSs extraction process. Macroalga drying and milling, followed by aqueous extraction, were examined at various temperatures, and their effects on the macroalga properties, on component extraction and extractability, and the degree of polymerisation of xylan/XOS, were analysed and compared.

### 2.3. Effect of Drying Temperature on P. palmata Colour

**Figure 1:**
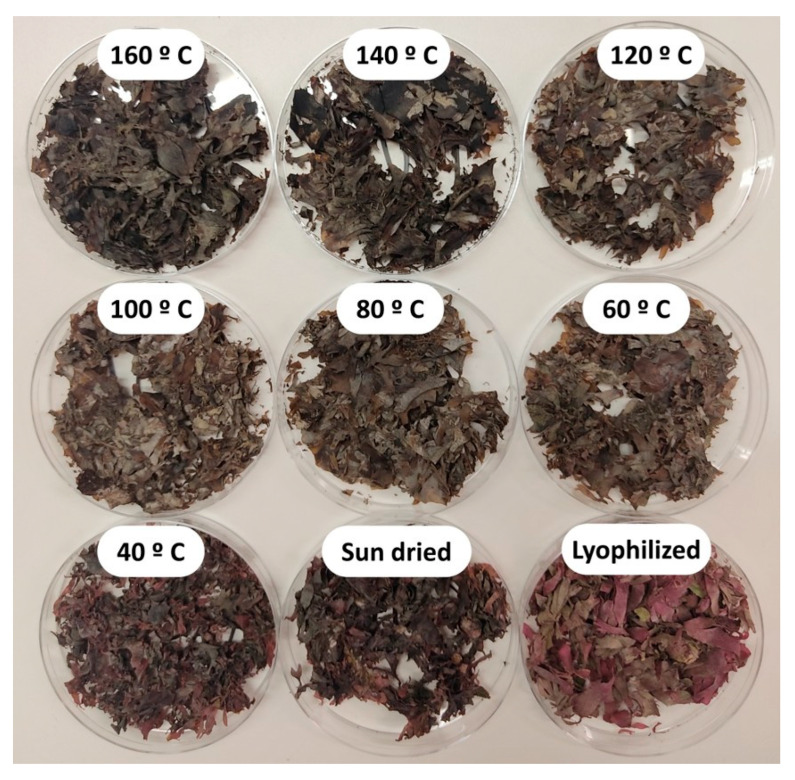
Visual aspects of *P. palmata* following lyophilisation, sun drying, and drying at temperatures from 40 to 160 °C.

Fresh algae were dried at temperatures from 40 to 160 °C, and the effects on these investigated. As controls, samples dried in the sun, or lyophilised, were also prepared. From Figure 1, it can be seen that sun-dried and lyophilised samples, as well as those dried at 40 or 60 °C, maintain their characteristic reddish colour, but at higher temperatures, this reddish colour is replaced by a dark green/grey colour. Indeed, at the medium temperatures tested (80–120 °C) green and brown become prevalent, while at higher temperatures (140–160 °C) black and grey tones are more noticeable.

The characteristic reddish colour of *P. palmata* is conferred by the phycobiliprotein phycoerythrin [63], a major light-harvesting protein–chromophore complex mainly found in cyanobacteria and red algae. Interestingly, the drying temperatures at which colour changes were seen for the macroalgae in our study, i.e., greater than 60 °C, correspond with the temperature reported in other studies as promoting loss of stability for this complex. B and R-phycoerythrin have been shown to be stable from 4 to 40 °C but display a significant loss of stability at 60 °C and total loss at 100 °C [64]. Another study reported stability for purified *P. palmata* R-phycoerythrin up to 60 °C, as no changes in colour nor fluorescence were observed up to this temperature, while at higher temperatures, absorption decreased, and eventually, above 90 °C, the solution turned colourless [65]. Thus, it is suggested that at the lower pretreatment temperatures investigated in our study, including drying at 40 and 60 °C, lyophilisation, and sun drying, phycoerythrin retained its overall structure and colour, and hence the dried algae maintained the reddish colour of the fresh biomass. At higher temperatures, however, above 60 °C, structural changes in the algae matrix and/or phycoerythrin complex occurred, leading to complex modification and/or even unfolding and/or disassembly and colour loss as seen in Figure 1.

### 2.4. Aqueous Extraction of Xylan

**Figure 2:**
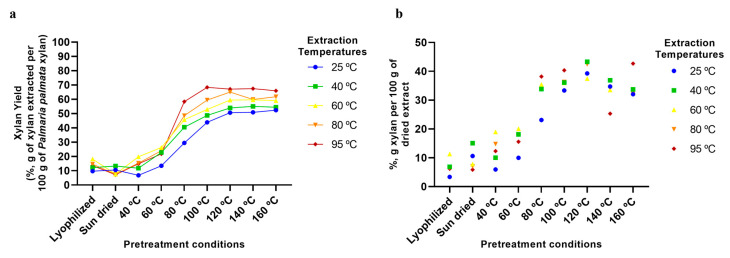
Xylan/xylo-oligosaccharides extraction using various pretreatment conditions and aqueous extraction temperatures: (**a**) Extraction yields of xylan from *P. palmata* (%, g of xylan extracted per 100 g of *P. palmata* xylan). (**b**) Xylan content of final dried extracts (%, g of xylan per 100 g of dried extract).

We next investigated the use of aqueous extraction at various temperatures for the extraction of xylan/XOS from the dried algae samples. Each of the samples dried at temperatures from 40 to 160 °C, as well as the sun-dried and lyophilised samples, were subjected to aqueous extraction for 2 h at temperatures ranging from 25 to 95 °C. From Figure 2a, it can be seen that temperature, both for seaweed drying and aqueous extraction, had a significant effect on the extractability of xylan from the macroalgae. Extraction yields from as low as 5 to 10% for the lyophilised, 40 °C dried, and sun-dried samples, to as high as 70% for the samples dried and extracted at the highest temperatures examined can be observed. Indeed, while a small increase in extractability occurred when the macroalgae was dried at 60 °C, the major change is seen at drying temperatures above 60 °C, similar to that observed for the change in alga colour. Extractability then stabilised at ~70% at 100 °C and higher drying temperatures. For aqueous extraction, the lowest yields are observed for 25 °C extractions and the highest yields for 95 °C extractions over the whole range of drying temperatures examined. Overall, the highest yields were obtained with processes wherein macroalgae were dried at temperatures from 100 to 160 °C and extracted at 95 °C (Figure 2a), leading to a final dried extract composed of ~40% xylan/XOS (Figure 2b).

**Figure 3:**
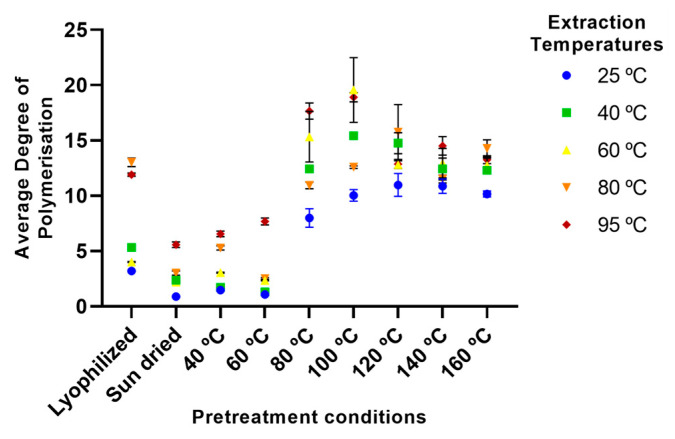
Average degree of polymerisation of xylan/xylo-oligosaccharides extracted using various pretreatment conditions and aqueous extraction temperatures. Error bars represent the standard deviation of the mean.

Investigation of the average degree of polymerisation (DP) of the extracted xylan (Figure 3), as determined from the relationship between the number of reducing ends and xylose monomers present, also showed a temperature dependence wherein an increase in average DP was observed with increasing drying temperatures, especially above 60 °C. Higher aqueous extraction temperatures also led to increased average DP, but this increase appears to be reduced for samples dried at the highest temperatures examined, i.e., 140 and 160 °C. Such observations suggest that preservation using low temperatures (40–60 °C), or sun drying, permit aqueous extraction of low amounts of shorter-chain xylan, with an average DP of ~1 to 8 being observed. On the other hand, more aggressive approaches, using higher temperatures, are required for the extraction of larger-sized products (average DP of ~20), while temperature extremes lead to decreased average DP, possibly as a result of product degradation [66].

### 2.5. Aqueous Extraction of P. palmata Components: Protein, Ash, Galactose, and Phenolic Compounds

**Figure 4:**
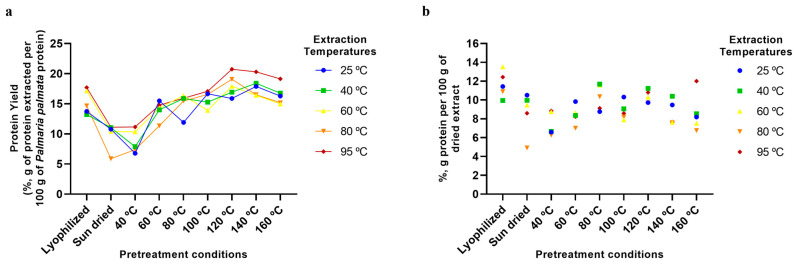
Protein extraction using various pretreatment conditions and aqueous extraction temperatures. (**a**) Extraction yields of protein from *P. palmata* (%, g of protein extracted per 100 g of *P. palmata* protein). (**b**) Protein content of final dried extracts (%, g of protein per 100 g of dried extract).

**Figure 5:**
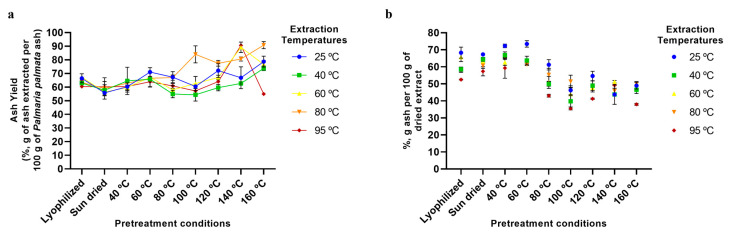
Ash extraction using various pretreatment conditions and aqueous extraction temperatures. (**a**) Extraction yields of ash from *P. palmata* (%, g of ash extracted per 100 g of *P. palmata* ash). (**b**) Ash content of final dried extracts (%, g of ash per 100 g of dried extract). Error bars represent the standard deviation of the mean.

**Figure 6:**
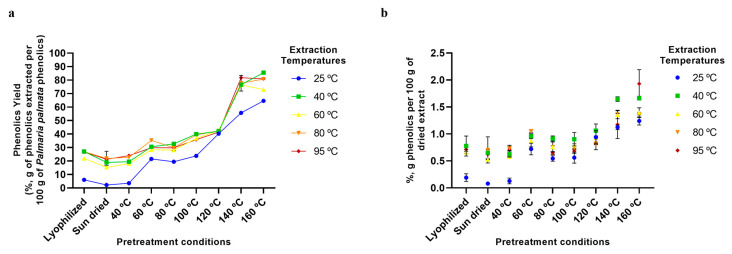
Extraction of phenolic compounds using various pretreatment conditions and aqueous extraction temperatures. (**a**) Extraction yields of phenolic molecules from *P. palmata* (%, g of phenolics extracted per 100 g of *P. palmata* phenolic compounds). (**b**) Phenolic compound content of final dried extracts (%, g of phenolics per 100 g of dried extract). Error bars represent the standard deviation of the mean.

**Figure 7:**
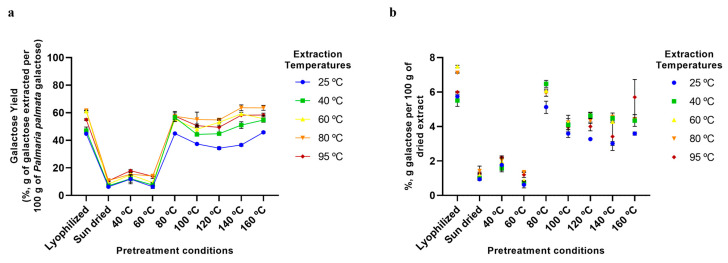
Galactose extraction using various pretreatment conditions and aqueous extraction temperatures. (**a**) Extraction yields of galactose from *P. palmata* (%, g of galactose extracted per 100 g of *P. palmata* galactose). (**b**) Galactose content of final dried extracts (%, g of galactose per 100 g of dried extract). Error bars represent the standard deviation of the mean.

The analyses of the extraction of other *P. palmata* components, namely protein (Figure 4), ash (Figure 5), galactose (Figure 6), and phenolics (Figure 7), confirmed the trend of higher yields with higher temperatures, especially for the drying temperature, but the yields observed and the extent of the increase in yields with temperature varied according to the component analysed. For protein, yields from as low as ~5% to a maximum of only 20% were observed (Figure 4a), with the majority of the protein being fractionated into the insoluble fraction, leading to a final dried soluble-xylan-rich extract composed of only ~9% protein (Figure 4b). This low yield in the soluble fraction, even at high treatment temperatures, is suggested to be a result of greater protein release from the algae matrix at higher temperatures being counteracted by protein precipitation and fractionation into the insoluble fraction. Furthermore, as previously mentioned, *P. palmata* protein demonstrates thermal instability at 60 °C and above, and this, in conjunction with the colour changes indicative of protein modification observed in our study following drying pretreatment at high temperatures (Section 2.3), suggests the protein in the higher temperature extracted soluble extracts to be in the denatured form.

Ash, on the other hand, showed yields from ~60 to ~90% (Figure 5a), resulting in this being a major component (~40–70%) of the final dried xylan-rich extract (Figure 5b).

The phenolic components also displayed a high extractability, up to a maximum of ~85% (Figure 6a), but due to the low initial concentrations of these, the final content in the extracts ranged from only ~0.1 to 2% (Figure 6b). *P. palmata* contains a variety of phenolic compounds, with phenolic acids (p-coumaric acid, gallic acid, and p-hydroxybenzoic acid), flavonoids, and bromophenols being most common. Importantly, these specific compounds are known to be particularly heat stable, up to greater than 200 °C in the case of some bromophenols and phenolic acids. In agreement with this, the positive results observed in our study with the Folin–Ciocalteu assay, which quantifies active molecules, suggests that those in our extracts, even those extracted at high temperatures, are intact, active phenolic compounds.

Similarly, low initial concentrations of galactose resulted in low amounts in the final extract (1–7%) (Figure 7b), even though extraction yields as high as ~60% were observed (Figure 7a).

No lipids were detected in the extracts with the protocols used, probably related to their low content in the macroalga studied and the tendency of water extraction to segregate hydrophobic compounds [67].

Finally, it can also be seen from Figure 2, Figure 3, Figure 4, Figure 5, Figure 6 and Figure 7 that the use of lyophilisation as a preservation treatment leads to variable extraction results, different from those of the drying methods examined. Depending on the component being extracted, somewhat similar or significantly higher extraction yields were observed than with the lower temperature and sun drying methods examined in our study. This observation reinforces our results, pointing to how the preservation approach used critically affects the extractability of each of the macroalgae components, and should be carefully designed to enhance extraction yields of the specific products of interest.

### 2.6. Effects of Biomass Concentration and Incubation Time on Extraction Process

**Figure 8:**
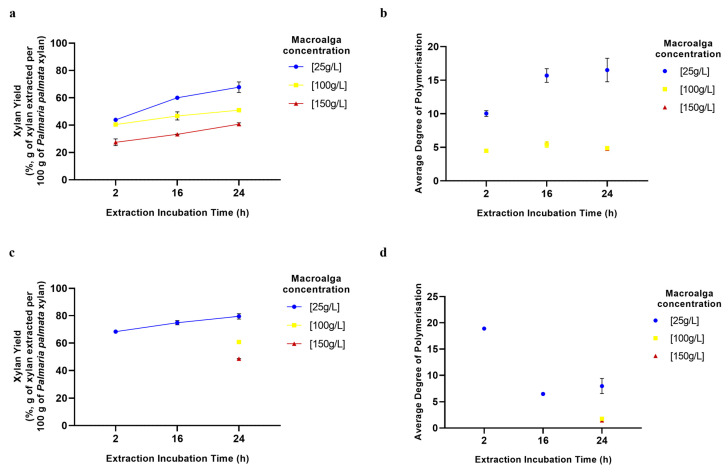
Effects of biomass concentration and incubation time on xylan/xylo-oligosaccharide yields and average degree of polymerisation. (**a**,**c**) Effects of extraction incubation time and macroalga concentration on xylan/xylo-oligosaccharide yields for extraction at 25 °C (**a**) and 95 °C (**c**). (**b**,**d**) Effects of extraction incubation time and macroalgae concentration on xylan/xylo-oligosaccharide average degree of polymerisation for extraction at 25 °C (**b**) and 95 °C (**d**). Error bars represent the standard deviation of the mean.

For investigation of the effects of the aqueous extraction process variables of dried macroalgae concentration and extraction time on final yields and product DP, we focused on two of the sequential extraction processes. Both employed macroalgae that were dried at 100 °C, but aqueous extraction was carried out either at 95 °C (highest xylan yield, ~70%) or 25 °C (reduced energetic cost, ~45% xylan extraction yield). As might be expected, higher macroalgae concentrations led to higher concentrations of total xylan/XOS extracted, but these were also found to have a negative effect on xylan yield (Figure 8a,c). Indeed, this trend in reduced extractability with increasing algae concentration was also observed for all other components and is probably due to a reduction in available water for component solubilisation at higher algae concentrations. In contrast, but also as might be expected, longer extraction times led to higher xylan/XOS yields at both extraction temperatures investigated (Figure 8a,c), thereby enabling maxima of as high as ~70% extraction at 25 °C and ~80% with a 95 °C extraction temperature. While similar trends were observed for the extraction of most of the other macroalgae components analysed, interestingly, a decrease in yield with increasing extraction time was observed for proteins, probably because of protein precipitation during extended incubation. Another interesting observation was the difference in average DP of extracted xylan depending on the extraction time and temperature used. Longer extraction times resulted in an increase in product average DP with a 25 °C extraction temperature (Figure 8b), but a decrease in product average DP was observed with a 95 °C extraction temperature (Figure 8d). It is suggested that in the first case, at 25 °C, longer extraction times enable better extraction of the more tightly bound longer xylan/XOS chains. On the other hand, in the case of the 95 °C extraction, it is suggested that extended incubation at this high temperature leads to product hydrolysis and lower DP products.

The high xylan yields achieved enabled for a final dried extract with ~40–50% xylan, low levels of proteins (~7–9%), and phenolic compounds (~0.5–1.2%), no detectable lipids, but also high ash/minerals concentrations (45–50%). *P. palmata* ash has been reported to consist mainly of Na, K, Ca, and Mg as well as microelements such as Fe, I, and Mn [49], all of which should be easily removed by, for example, dialysis or ultrafiltration, or with desalting columns. We showed that dialysis in water with a 100–500 Dalton molecular weight cut-off membrane and drying, provided a final dried product with no detectable ash and consisting of as high as 93 ± 0.41% xylan, 7 ± 0.47% protein, and 0.45 ± 0.008% phenolic compounds. Such a high product purity (~90%) combined with the high xylan/XOS yields attained (~80%) makes this simple, sequential xylan/XOS extraction process one of the most effective methods reported to date. This process will promote the exploitation potential of these compounds, stimulating their more widespread use across a wider spectrum of applications. Importantly also, these xylan/XOS compounds are water soluble and display non-branched, mixed-linkage (β-1,3/β-1,4-linked) structures which differ from the branched, β-1,4-linked structures of currently used xylans/XOS. Such novel structures should display altered and/or novel functions, bioactivities, and/or applications, and further studies will focus on investigating this.

Currently reported xylan yields vary from ~5 to 50%, with the majority being extracted from terrestrial lignocellulosic plant material via multi-step procedures employing hazardous chemicals (e.g., acids, alkali, ammonia, sodium hypochlorite, alkaline peroxide, and solvents treatments), often combined with harsh physical processes (e.g., hydrothermal treatment, ultra-high temperatures, high pressures, and steam blasting) and/or enzyme treatments requiring specific reaction conditions, equipment and expertise. Such processes are characterised by high environmental and economic costs and often lead to impure products and undesired and sometimes hazardous waste and side products. Importantly, a xylan extraction method has already been described [68] for a red macroalga identified as a Japanese *Palmaria* sp., but which is more probably *Devaleraea inkyuleei* [69]. The described multi-step process involves lyophilisation, grinding, washing twice in chloroform–methanol solution and in acetone, with filtration between each step, resuspension in water and autoclaving at 121 °C for 20 min, extraction with 8 M urea, filtration, dialysis against water, centrifugation, and lyophilisation [68]. This process led to a xylan extraction yield of 33.8% and final product purity of 52%. It can be seen how our process offers advantages over the currently described processes, being a simpler, more sustainable extraction process with reduced environmental and economic costs as well as much higher yields and a higher final product purity. The success of our process is based on the careful selection of the temperature used for the preservation step, with, depending on the temperature employed, this drying process variably promoting modifications and structural alterations within the algae structures that affect the extractability of the algae components. Therefore, it is reinforced [60] that the preservation step of macroalgae production processes should be seen as not only being important for algae storage, but also as being an important part of, or a pretreatment step in, the extraction processes.

## 3. Materials and Methods

### 3.1. P. palmata Biomass Preparation and Preservation Treatments

Fresh *P. palmata* ((Linnaeus) F. Weber & D. Mohr, 1805) biomass was provided by ALGAplus, ÍIhavo, Portugal (40°36′44.7″ N, 8°40′27.0″ W). The seaweed was cultivated in a land-based integrated multi-trophic aquaculture (IMTA) system, where fish farm effluent is used to increase the availability of dissolved inorganic nitrogen for seaweed growth. In the present study, it was harvested in May 2023, cleaned to remove epiphytes, washed with filtered seawater, and divided into 200 g batches. Each batch was then immediately subjected to one of the following preservation processes: lyophilisation, sun drying, or oven drying at 40 °C, 60 °C, 80 °C, 100 °C, 120 °C, 140 °C, or 160 °C. For lyophilisation, the algae sample was frozen at −80 °C overnight and lyophilised in a Christ-Alpha 2–4 LD lyophiliser until weight stabilisation (70 h). For sun drying, the algae sample was placed outside in an oven tray with direct sun exposure until complete weight stabilisation (without further water loss), and with the sample being stored during the night at room temperature before resumption of sun drying the following day. For oven drying, the algae samples were placed on oven trays, covered with perforated aluminium foil, and dried in ovens at the respective temperature until complete stabilisation of weight. Following treatment, all samples were milled using a laboratory blender (8011G, Waring Laboratory Science, Stamford, CT, USA) at maximum speed (22,000 rpm) with 2 min pulses until the sample had reached a particle size small enough to pass through a 200 μm sieve. All samples were stored in opaque containers at room temperature until use.

### 3.2. Compositional Analysis of P. palmata

A complete compositional analysis of *P. palmata* was performed using lyophilised samples. All analyses were carried out in triplicate (*n* = 3). The moisture and ash content were determined with the thermal gravimetric analysis methods numbers 950.46 and 938.08 [70], respectively. Briefly, for moisture content determination, 1 g of dried sample was incubated at 80 °C until weight stabilisation, and reweighed. For ash determination, 1 g of dried sample was incubated at 550 °C for 16 h in a muffle furnace (Nabertherm LVT 15/11) and then reweighed. The lipid content was determined by the Bligh and Dyer method [71]. For this, 2 g of lyophilised samples were mixed with 0.8 volumes of water, 1 volume of chloroform, and 2 volumes of methanol by vortexing. One volume of chloroform was then added and the suspension again vortexed, before the addition of one volume of distilled water and stirring for 10 min. The mixture was filtered through a grade 1 paper filter (Whatman), transferred to a graduated cylinder, and left to stand until complete phase separation and clarification occurred. The volume of the lower chloroform layer containing the purified lipids was recorded, and the upper layer was removed by aspiration. Five samples of 1 mL of the chloroform layer were then evaporated at room temperature overnight in a fume hood in pre-weighed tubes, and the lipid content calculated from sample weights as described in [71]. The crude protein content was measured with the micro-Kjeldahl method number 988.05 [70]. A mass of 0.2 g of lyophilised sample was digested with 1 g of Kjeldahl catalyst and 4 mL of H_2_SO_4_ at 400 °C for 2 h, and the reaction was stopped by the addition of 20 mL of deionised water. Samples were distilled using 30 mL of 10 M NaOH, with a boric acid–bromocresol-methyl red solution as indicator. The resulting solution was then titrated with 0.1 M HCl, and the total nitrogen and protein contents calculated according to method number 988.05 [70] with the use of a protein factor of 5 [50]. The total phenolic content was determined using an adapted Folin–Ciocalteu method [50,72]. Briefly, phenolic compounds were first extracted from 0.5 g of biomass by four consecutive extractions with 4 mL of distilled water at 98 °C for 5 h, and then pooled and mixed. The extracts were centrifuged at 5000 rpm for 10 min, and 20 µL of supernatant was mixed with 1580 µL distilled water before the addition of 100 µL 2 N Folin reagent (Thermo Fisher Scientific) and incubation for 5 min at room temperature in the absence of light. A 300 µL volume of freshly prepared 2 M Na_2_CO_3_ was then added and incubated for an additional 60 min at room temperature in the absence of light. The absorbance at 750 nm was measured and the phenolic content calculated from a gallic acid standard curve. For carbohydrate content determination, algae samples were first submitted to four consecutive H_2_SO_4_ and autoclave hydrolysis and extraction treatments before extract analysis by HPLC. For this, 1 g of dried algae was suspended in a final volume of 10 mL 4% (*w*/*v*) H_2_SO_4_ and treated at 121 °C, 1 bar pressure for 20 min. Hydrolysed samples were centrifuged at 9000 rpm for 30 min and filtered through a 0.22 µm polyethersulfone (PES) filter (Merck). The supernatant was retained, and the pellet submitted to three further H_2_SO_4_ and autoclave hydrolysis and extraction treatments as described above, and all supernatants were pooled and analysed by HPLC. HPLC was carried out with a ROA organic acid H+(8%) column (Phenomenex) at 60 °C, using an Elite LaChrom (VWR Hitachi) chromatography system with an Elite LaChrom L-2490 RI detector (VWR Hitachi) at 40 °C. A 2.5 mM H_2_SO_4_ mobile phase at a flow rate of 0.7 mL/min for the first 7 min, followed by 0.1 mL/min for a total period of 30 min, was used. The EZChrom Elite 3.3.2 SP2 software was employed for data collection and analysis. Sugar monomer concentrations were calculated from the respective standard curves of each monomer investigated (xylose, glucuronic acid, glucose, and arabinose). The presence of cellulose, as a fibrous material, was analysed using the two-step hydrolysis protocol for glucose extraction as described in [73], and HPLC analysis of the extracts was performed as described above. The galactose content was determined using the arabinose–galactose quantification kit (K-Arga) according to the manufacturer’s (Megazyme, Bray, Ireland) instructions. For sample preparation, 100 mg of sample was hydrolysed with 5 mL of 1.3 M HCl at 100 °C for 1 h and then neutralised with 5 mL of 1.3 M NaOH. The solution volume was then adjusted to 100 mL with distilled water and mixed thoroughly, and 100 µL samples were centrifuged at 1500× *g* for 10 min, and the supernatant analysed according to the kit assay procedure described in the manufacturers instructions. The absence of arabinose was confirmed by HPLC on a ROA organic acid H+(8%) column (Phenomenex) as described above for the carbohydrate content determination.

### 3.3. Aqueous Extraction of Xylan

For examination of the aqueous extraction of xylan, each of the macroalgae samples subjected to the preservation treatments described above were incubated at a concentration of 25 g/L (500 mg in 20 mL distilled water in a 100 mL Schott flask) in a GFL 1083 shaking water bath (GFL, Burgwedel, Germany) for 2 h at 120 rpm at the following extraction temperatures: 25 °C, 40 °C, 60 °C, 80 °C, or 95 °C. For the macroalgae concentration and extraction time optimisation, 100 °C oven dried *P. palmata* was subjected to aqueous extraction at 25 °C or 95 °C, with macroalgae concentrations of 25, 100, or 150 g/L, and extraction times of 2, 16, or 24 h. Following extraction, macroalgae suspensions were transferred to pre-weighted centrifuge tubes, and the extraction flasks were washed with 1 mL of distilled water that was then also added to the suspensions. The suspensions were centrifuged at 9000× *g* at room temperature for 30 min, and the supernatant (soluble fraction) was separated and collected. The pellet (insoluble fraction) was washed by vortexing with 1 mL of distilled water, centrifuged at 9000× *g* for 30 min at room temperature, and the supernatant was pooled with the suspension supernatant. The insoluble and soluble fractions were frozen at −80 °C, lyophilised, and stored in sealed containers at room temperature until further analysis. The remaining soluble fraction was immediately analysed as described below. All extractions were performed in duplicate (*n* = 2), and the optimisation assays were carried out in triplicate (*n* = 3).

### 3.4. Compositional Analysis of the Extracts

The composition of the soluble fraction was determined in order to understand how the different components are affected differently by the preservation and extraction conditions examined and to assess the purity grade of the extracts. All assays were carried out in triplicate (*n* = 3). The pH of the soluble fraction was measured with an Edge pH electrode (Hanna Instruments, Woonsocket, USA). For xylan concentration determination, 2 mL of samples were mixed with H_2_SO_4_ at a final concentration of 4% (*w*/*v*) and autoclaved at 121 °C and 1 bar pressure for 20 min. The hydrolysates were centrifuged for 30 min at 5000× *g* at room temperature, and the supernatant filtered through a 0.22 µm polyethersulfone filter (Merck) before HPLC analyses as described above (Section 3.2). Xylose concentrations were calculated from the respective standard curve. The galactose content was measured using the arabinose–galactose quantification kit according to the manufacturer’s (Megazyme, Bray, Ireland) instructions. Phenolic compounds in the soluble fraction were determined as described in Section 2.2 above using 20 µL of sample. The soluble protein content was measured by the modified Lowry method as proposed in [74]. Briefly, 5 mg of lyophilised soluble fraction was solubilised in 1 mL of reagent D (48:1:1 of 2% (*w*/*v*) anhydrous Na_2_CO_3_ in 0.1 M NaOH; 1% (*w*/*v*) NaK tartrate tetrahydrate; 0.5% (*w*/*v*) CuSO_4_ pentahydrate) for 3 h at 55 °C and 1000 rpm in a SC-18/02C Thermo-shaker (BioSan, Riga, Latvia). Following cooling at room temperature, samples were centrifuged at 15,000× *g* for 15 min at room temperature, and 20 µL of supernatant was added to 980 µL of reagent D and incubated for 10 min at room temperature in the absence of light. A volume of 100 µL of Folin–Ciocalteu reagent (1 N) was then added, the samples were vortexed immediately, incubated at room temperature for 30 min, and the absorbance was measured at 750 nm. Bovine serum albumin (BSA) at concentrations ranging from 250 to 1500 µg/mL was used for the standard curve. The lipid and ash contents of the lyophilised soluble fraction were assessed, respectively, by the Bligh and Dyer method [71] and method no. 938.08 [70].

### 3.5. Analysis of the Degree of Polymerisation of Xylan

The average degree of polymerisation (avDP) of xylan was calculated as follows:

$$avDP\;=\frac{Total\;\left[xylose\;in\;sample\right]}{Total\;\left[reducing\;ends\right]}$$

To quantify the concentration of reducing ends present in the extracts, the 3,5-dinitrosalicylic acid (DNSA) method [75] was employed. Xylose was used as the standard. Following mixing of 0.25 mL of the xylose standards or the liquid soluble fraction to be analysed with 0.5 mL of DNS reagent, samples were heated at 100 °C for 10 min. The samples were then cooled on ice for 5 min, and the absorption at 546 nm was measured.

The concentration of xylose in the soluble fraction was analysed by HPLC following H_2_SO_4_ and autoclave treatment as described in Section 3.4 above.

## 4. Conclusions

In this study, we developed the five-step protocol shown in Equation (1) for the simple, high-yield extraction of highly pure xylan/XOS from the red macroalga *P. palmata*.

Oven Drying (≥100 °C)—Milling—Aqueous Extraction—Centrifugation—Dialysis.(1)

Equation (1) Process for high-yield xylan/XOS extraction from *P. palmata*. We show that success with this process is critically dependent on the preservation process employed, with oven drying, especially at high temperatures, i.e., at 100 °C or above, enabling high final xylan yields and purity. Extraction yields increase with drying and extraction temperatures, with a major transition being observed at drying temperatures of approximately 60–80 °C, as evidenced by a change in algae colour, an increase in component yield, especially xylan yield, and an increase in the extracted xylan degree of polymerisation. At oven drying temperatures of 100 °C and above, a plateau is reached, with no further or only minor changes being observed in most cases. These observations suggest that at ~60 °C and higher drying temperatures, structural changes are induced in the algae matrix, including protein unfolding, which facilitate the subsequent aqueous extraction of the soluble xylan/XOS via separation of the majority of the protein into the insoluble fraction and the xylan/XOS into the soluble fraction. We chose 100 °C as the optimal drying temperature as this enables the highest xylan/XOS yields, while reducing energy requirements associated with higher drying temperatures. For the aqueous extraction step, we showed that both low (25 °C) and high (95 °C) temperatures are effective. Higher extraction temperatures permit higher final xylan extraction yields (up to 90%), but high yields (up to 80%) are also observed with low-temperature extraction, especially with longer extraction times, up to 24 h.

Following aqueous extraction, we used centrifugation to separate the xylan-rich soluble fraction from the protein-rich insoluble fraction, and dialysis of the supernatant to remove the high levels of ash present. Neither glucose nor lipids were detected in the extracts, but low amounts of polyphenolic compounds and galactose were co-extracted with the xylan. Nevertheless, the initial concentrations of these in *Palmaria palmata* are low and therefore their final content in the xylan-rich extract is low. Thus, our simple process, consisting of 100 °C oven drying of fresh macroalgae, milling, aqueous extraction with 25 g/L dried milled algae at 95 °C for 24 h, centrifugation, and dialysis, enables a yield as high as 80% and a highly pure product composed of as much as 90% xylan/XOS. Importantly, the use of a 25 °C aqueous extraction temperature with 24 h extraction also gives rise to high yields, up to 70%, this being higher than many currently reported xylan/XOS extraction yields as discussed above, and potentially enables reduced economic and environmental costs of the process.

Importantly, our process not only gives rise to a xylan-rich soluble fraction but also a protein-rich insoluble fraction composed of approximately 60% protein (for the 95 °C extraction process). As the world’s populations turn towards new sources of proteins and bioactive compounds, such as bioactive peptides, for healthier lifestyles and health care, it can be seen that this protein-rich fraction can also be of much value. Indeed, the exploitation of both fractions, i.e., the xylan-rich and protein-rich fractions, will enable a multi-valorisation of *P. palmata*, enhancing the value of this marine biomass, and contributing to the circular and bio-based economy concepts by valorising the principal algal components and reducing wastes with production of novel bioactive compounds.

## Data Availability

The original data presented in this study are included in the article; further inquiries can be directed to the corresponding author.

## Abbreviations

The following abbreviations are used in this manuscript:

DNSA	3,5-dinitrosalicylic acid
XOS	Xylo-oligosaccharides
HPLC	High-Performance Liquid Chromatography
IMTA	Integrated Multi-Trophic Aquaculture
DP	Degree of Polymerisation
PES	Polyethersulfone
RI	Refractor Index
BSA	Bovin Serum Albumin
